# Congestive heart failure in cattle; etiology, clinical, and ultrasonographic findings in 67 cases

**DOI:** 10.14202/vetworld.2020.1145-1152

**Published:** 2020-06-19

**Authors:** Mustafa Abd El Raouf, Magdy Elgioushy, Shimaa A. Ezzeldein

**Affiliations:** 1 Department of Surgery, Anesthesiology and Radiology, Faculty of Veterinary Medicine, Zagazig University, Zagazig, 44519, Egypt; 2 Department of Animal Medicine, Faculty of Veterinary Medicine, Aswan University, Aswan 37916, Egypt

**Keywords:** cattle, congestive heart failure, mediastinal abscess, pericarditis, pleurisy

## Abstract

**Background and Aim:**

Congestive heart failure (CHF) is a clinical disorder that results from cardiac dysfunction with subsequent fatal outcomes in most cases. Several diseases are incriminated in occurrence of CHF. Therefore, the aims of this study were to identify CHF etiology and associated clinical findings in 67 cows and to investigate the relationship between CHF and the other body organs using ultrasonographic examination.

**Materials and Methods:**

Sixty-seven cows affected by CHF admitted to the clinic with a history of loss of appetite, decrease in milk production, constipation, and brisket edema were thoroughly investigated clinically and ultrasonographically. In addition, ten apparently healthy cows were used as a control group.

**Results:**

Clinically, cows with CHF manifested jugular engorgement and pulsation (88.1%), brisket and/or intermandibular edema (77.6%), and muffled heart sounds (76.1%). Based on the ultrasonographic examination, traumatic pericarditis (82.1%) was the most prevalent etiology of CHF. Extracardiac etiology of CHF identified were exudative pleurisy (10.4%) and mediastinal abscesses (7.5%). Hepatomegaly (88.1%) and pleural effusion (61.2%) were the most documented consequences.

**Conclusion:**

Both cardiac and extracardiac diseases could be associated with CHF in cattle. Ultrasonographic changes in liver and pleura secondary to CHF were the most common findings. Ultrasonography is a good tool for the diagnosis of cardiac and extracardiac etiologies of CHF in cattle.

## Introduction

Congestive heart failure (CHF) is the end-stage of cardiac disease at which point compensatory mechanisms are disrupted, leading to adverse effects on the myocardium and cardiac output [1,2]. The clinical signs of CHF in cattle vary according to the severity of the disease and the extent of increasing hydrostatic pressure. The most reported signs include syncope, weakness, intolerance to exercise, jugular vein distension and pulsation, and subcutaneous edema. Cardiac arrhythmias and cardiac murmur could be recorded in addition to muffled heart sounds [3]. In cattle, traumatic pericarditis (TP) due to foreign body penetration through the reticulum is considered the main cause of CHF [4-8]. Extension of infection to the pericardium from pleural or lung infections [9], tumors such as lymphoma [10], or idiopathic pericarditis [11] are also possible causes.

Although the clinical findings of CHF are diagnostic, several animals may not exhibit the characteristic signs [4,5,12]. Field diagnosis of such cases is very important and provides prognostic value for the animals avoiding unnecessary treatment and costs. Ultrasonography is a rapid, accurate, and low-cost diagnostic tool for heart diseases that can be easily done in the field environment with high sensitivity and specificity [5,13,14].

The aims of the present study were to identify different CHF etiology and associated clinical findings in 67 cows and to investigate the relationship between CHF and the other body organs using ultrasonographic examination.

## Materials and Methods

### Ethical approval

The present study was conducted according to the animal use welfare and ethical committee of Zagazig University, Faculty of Veterinary Medicine, Egypt.

### Animals and clinical examination

Sixty-seven cows aged 3-7 years and weighed 300-550 kg were admitted to the hospital of the Faculty of Veterinary Medicine – Zagazig University during the period from June, 2018 to December, 2019 with a history of partial to complete loss of appetite, loss of body weight, decreased milk production in lactating cows, constipation, tympany, abducted elbow joint, jugular engorgement, and brisket edema. Treatment attempts by field veterinarians were failed. Ten apparently healthy cows were used as a control group for clinical and ultrasonographic examinations.

All cows were thoroughly examined clinically as reported previously by Rosenberger [15]. Heart rate and sounds, rectal body temperature, respiratory rate and lung sounds, mucous membranes, and ruminal movement were reported. Foreign body test using metal detector and pain tests for the heart and lungs were performed for each animal.

### Ultrasonographic examination

Ultrasonographic scanning of the thorax and abdomen of the healthy and affected animals was carried out in a standing position using an ultrasound machine (SonoScape A5V, China) connected with 3.5 MHZ convex transducer. The areas from 3^rd^ to 12^th^ intercostal spaces (ICS) at both sides of the thorax and the abdomen were prepared for ultrasonographic examination by hair shaving and ultrasound coupling gel application.

Thoracic ultrasonography was performed following standardized examination methods, as reported previously by Mohamed [16]. Each lung was examined by firm placing the transducer parallel to the ribs at each ICS extending from 3^rd^ to 11^th^ ICS to evaluate the pleura, pulmonary tissue, pleural effusion, lung consolidation, and comet tail artifacts. Cardiac ultrasonography was performed according to previously described methods [17]. The heart was examined at the right thorax, then the left side at the 3^rd^ to 5^th^ ICS to evaluate the pericardium, myocardium, endocardium, and the presence of fluid and/or fibrin inside the pericardial sac.

Abdominal ultrasonography was performed following the previous study [18]. The reticulum, rumen, spleen, liver, kidney, omasum, abomasum, intestine, and peritoneum were examined for any alteration in their normal structure and movement. The dimensions of the liver, portal vein (PV), and caudal vena cava (CVC) were measured as described previously [13,19], while the spleen was examined as previously described [19].

### Confirmatory tests

Ultrasonographic-guided abdominocentesis [20] was performed in ascitic fluid suffering cases. Briefly, a sterile stainless steel needle was inserted through the abdominal wall at the right caudoventral quadrant of the abdomen after aseptic preparation of the penetration site under the guidance of ultrasonography. Ultrasonographic-guided thoracentesis [21] was performed in cases that suffered with thoracic lesions. Briefly, a sterile stainless steel needle was inserted through the thoracic wall at the level of the left 5^th^ ICS after aseptic preparation of the penetration site under the guidance of ultrasonography.

### Statistical analysis

The statistical analysis was done using the Kruskal–Wallis test with *post hoc* Dunn’s multiple comparison test because of the small size of the control group. The results are presented as mean±standard deviation. Differences between the control and diseased groups were considered statistically significant at p<0.05. Data were analyzed using Stata version 13 (Stata Corp., College Station, Texas, USA).

## Results

Based on the ultrasonographic findings, the affected cows were diagnosed with CHF accompanying TP (n=55), exudative pleurisy (n=7), and mediastinal abscess (n=5).

### Clinical findings

Jugular vein engorgement and pulsation (88.1%), brisket and/or intermandibular edema (77.6%), abduction of the elbow joint (67.2%), abnormal heart sounds (100%), tachycardia (67.2%), and ruminal stasis (100%) were the most reported clinical findings in the affected cows (Table-1).

**Table-1:** History and clinical findings of the cattle affected by CHF.

Items	Causes of CHF in cattle (n=67)			

	TP (n=55)	Pleurisy (Exudative stage) (n=7)	Mediastinal abscess (n=5)	Total (%)
		
	No. of animals (%)	No. of animals (%)	No. of animals (%)	
Loss of appetite	55 (100)	7 (100)	5 (100)	67 (100)
Decrease in milk production	38 (100)*	3 (100)*	3 (100)*	44 (100)*
Rectal temperature				
>39.2	14 (25.4)	5 (71.4)	3 (60)	22 (32.8)
38-39.2°C	32 (58.2)	0	2 (40)	34 (50.7)
<38	9 (16.4)	2 (28.6)	0	11 (16.4)
Respiratory rate/min >30	15 (27.3)	7 (100)	2 (40)	24 (35.8)
Heart rate/min >80 bpm	35(63.6)	5 (71.4)	5 (100)	45 (67.2)
Ruminal stasis	55 (100)	7 (100)	5 (100)	67 (100)
Tympany	25 (45.5)	0	0	25 (37.3)
Scanty hard feces	35 (63.6)	5 (71.4)	5 (100)	45 (67.2)
Weight loss	39 (70.9)	2 (28.6)	4 (80)	45 (67.2)
Congestion of the mucous membrane	47 (85.45)	6 (85.7)	5 (100)	58 (86.56)
Arched back	35 (63.6)	0	0	35 (52.2)
Recumbency	7 (12.7)	1 (14.3)	0	8 (11.9)
Heart sounds				
Splashing	11 (20)	0	0	11 (16.4)
Tinkling	5 (9)	0	0	5 (7.5)
Muffled	39 (70.9)	7 (100)	5 (100)	51 (76.1)
Abnormal lung sounds	32 (58.2)	7 (100)	2 (40)	41 (61.2)
Dyspnea	32 (58.2)	7 (100)	2 (40)	41 (61.2)
Cough	14 (25.5)	7 (100)	2 (40)	23 (34.3)
Jugular engorgement	47 (85.5)	7 (100)	5 (100)	59 (88.1)
Brisket and/or intermandibular edema	43 (78.2)	3 (42.9)	3 (60)	52 (77.6)
Elbow abduction	33 (60)	7 (100)	5 (100)	45 (67.2)
Positive foreign body test	48 (87.3)	0	0	48 (71.6)
Positive pain tests	35 (63.6)	7 (100)	2 (40)	44 (65.7)

* milking animals. CHF=Congestive heart failure, TP=Traumatic pericarditis

#### TP (n=55)

The most documented clinical findings were jugular engorgement and pulsation (85.5%), brisket and/or intermandibular edema (78.2%), and abnormal heart sounds (100%). With concern to the heart sounds, muffled (70.9%), splashing (20%), and tinkling (9%) sounds were reported in the affected cows. On auscultation, tachycardia was recorded in 63.6% of the affected animals. Tympany and scanty hard feces were reported in 45.5% and 63.6% of the affected animals, respectively. Arched back and positive pain tests were reported in 63.6% of the affected animals. Most of the affected cows (87.3%) expressed positive foreign body test using the metal detector. Abnormal lung sounds and dyspnea were documented in 58.2% of the affected cows. The cough was also reported in 25.5% of the affected cows. Recumbency and subnormal rectal body temperature were reported in 12.7% and 16.4% of the affected cows, respectively.

#### Pleurisy (n=7)

Dyspnea, cough, abduction of the elbow joint, jugular engorgement and pulsation, muffled heart sounds, abnormal lung sounds, and positive pain tests were the most documented clinical findings in all cases. Tachycardia, brisket and/or intermandibular edema, and fever were documented in 71.4%, 42.9%, and 71.4% of the affected cows, respectively. Subnormal rectal body temperature and recumbency were reported in 28.6% and 14.3% of the affected cows, respectively.

#### Mediastinal abscess (n=5)

Jugular engorgement, elbow abduction, muffled heart sounds, and tachycardia were the most reported clinical findings in the affected animals. Brisket and/or intermandibular edema, and fever were documented in 60% of the affected cows. Abnormal lung sounds, dyspnea, and cough were also manifested in 40% of the affected animals.

### Ultrasonographic findings

Ultrasonographic findings of the clinically healthy and CHF affected cows are summarized in Table-2. The underlying disease of CHF identified was TP (82.1%), pleurisy of the exudative stage (10.4%), and the mediastinal abscess (7.5%).

**Table-2:** Ultrasonography of the clinically healthy and CHF affected cattle.

Items		Clinically healthy cattle (n=10)	Causes of CHF (n=67)			

			TP (n=55)	Pleurisy (Exudative stage) (n=7)	Mediastinal abscess (n=5)	Total (%)
			No. of animal (%)	No. of animal (%)	No. of animals (%)	
Heart	Normal	10 (100)	0	5 (71.4)	3 (60)	8 (11.9)
	Fibrinous pericarditis	0	20 (36.4)	0	0	20 (29.9)
	Fibrino- suppurative pericarditis	0	17 (30.9)	0	0	17 (25.4)
	Suppurative pericarditis	0	18 (32.7)	2 (28.6)	2 (40)	22 (32.8)
Lungs and pleura	Pleural effusion	0	32 (58.2)	7 (100)	2 (40)	41 (61.2)
	Lung consolidation	0	5 (9.1)	7 (100)	2 (40)	14 (20.9)
Reticulum	Reticular contractions/3 min.					
	3	10 (100)	0	0	0	0
	2	0	21 (38.2)	5 (71.4)	4 (80)	30 (44.8)
	1	0	19 (34.5)	2 (28.6)	1 (20)	22 (32.8)
	0	0	15 (27.3)	0	0	15 (22.4)
	Half-moon shaped with smooth surface	10	0	7 (100)	5 (100)	12 (17.9)
	Reticular wall corrugation	0	27 (49.1)	0	0	27 (40.3)
	Reticular adhesions	0	21 (38.2)	0	0	21 (31.3)
	Reticular abscess	0	7 (12.7)	0	0	7 (10.4)
Liver	Liver size*	33.1±2.18^c^	46.3±2.21^a^	39.8±1.5^b^	40.2±1.6^b^	-
	PV*	3.64±0.16^c^	4.53±0.29^a^	4.02±0.15^b^	4.08±0.19^b^	-
	CVC*	3.25±0.2^d^	4.71±0.29^a^	3.5±0.19^c^	4.04±0.18^b^	-
	Liver angle					
	Acute	10	3 (5.5)	3 (42.9)	2 (400)	8 (11.9)
	Slightly acute	0	13 (23.6)	2 (28.6)	1 (20)	16 (23.9)
	Round	0	39 (70.9)	2 (28.6)	2 (40)	43 (64.2)
Spleen	Splenomegaly	0	5 (9.1)	0	0	5 (7.5)
Ascites		0	29 (52.7)	1 (14.3)	1 (20)	31 (46.3)

* The lowercase letters (a,b,c) within rows are significantly different (p<0.05). CHF=Congestive heart failure, TP=Traumatic pericarditis, PV=Portal vein, CV=Caudal vena cava

#### Clinically healthy animals (n=10)

**Figure-1:**
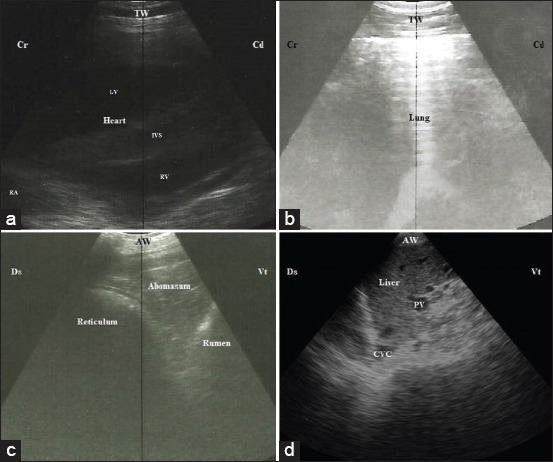
Normal ultrasonographic imaging of the thorax and the abdomen of a control cow. (a) The normal heart imaging scanned at the level of 4^th^ intercostal space (ICS); caudal long axis of the heart, left side. (b) The normal lung with its reverberation artifacts. (c) The reticulum appeared half-moon shaped echogenic wall with its neighboring organs including ventral blind sac of the rumen and the abomasum when scanned at 7^th^ ICS. (d) The liver appeared as homogenous echogenic structure with triangular-shaped CVC when scanned at the level of 11^th^ ICS. TW=Thoracic wall, LV=Left ventricle, IVS=Interventricular septum, RV=Right ventricle, RA=Right atrium, AW=Abdominal wall, PV=Portal vein, CVC=Caudal vena cava, Cr=Cranial, Cd=Caudal.

Ultrasonography of the thorax revealed a normal heart that appeared as four chambers with normal hypoechogenic cardiac muscle and two echogenic lines of the pericardium without effusion (Figure-1a). The pleura appeared as two echogenic lines under the thoracic wall representing parietal and visceral pleura without effusion. The normal lungs were characterized by normal reverberation artifacts (Figure-1b). Abdominal ultrasonography revealed half-moon shaped reticulum with a smooth surface and regular biphasic contractions 3 times in 3 min. Spleen, craniodorsal, and ventral sacs of the rumen, abomasum, and diaphragm were the neighboring organs to the reticulum and appeared without any abnormal changes in their structure or echogenicity (Figure-1c). The spleen could be visualized from 7^th^ to 12^th^ ICS with a tapered ventral end. The liver appeared as a homogenous echogenic parenchyma with an acute angle. The liver size was variable at each ICS and decreased cranially due to the superimposition of the lungs. The largest liver size was recorded at 10^th^ to 12^th^ ICS. The PV is characterized by its stellate ramifications with echogenic wall and can be visualized at 8^th^ to 12^th^ ICS. The CVC could be visualized at 11^th^, 12^th^ ICS, and rarely at 10^th^ ICS with its characteristic triangular shape in the cross-section (Figure-1d).

#### TP

**Figure-2:**
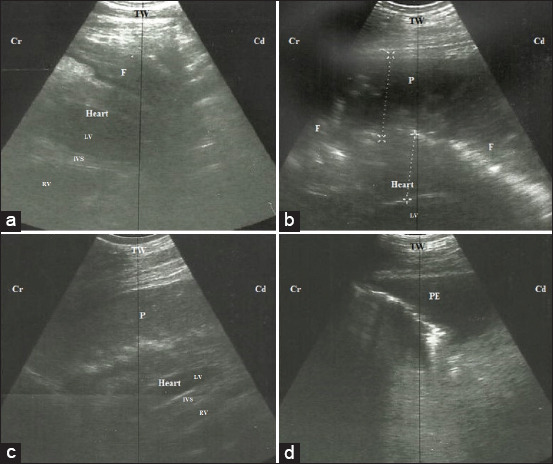
Ultrasonographic imaging of the heart scanned at the level of 4^th^ intercostal space of three cattle suffered from congestive heart failure due to traumatic pericarditis; caudal long axis of the heart, left side. (a) Accumulation of hyperechogenic exudate inside the pericardium representing fibrinous pericarditis. (b) Accumulation of anechoic pus inside the pericardium with finger like hyperechogenic fibrin strands representing fibrino-suppurative pericarditis. (c) Accumulation of hypoechogenic pus inside the pericardium representing suppurative pericarditis. (d) Presence of anechogenic exudate inside the pleura. TW=Thoracic wall, LV=Left ventricle, IVS=Interventricular septum, RV=Right ventricle, F=Fibrin strands, P=Pus, PE=Pleural effusion, Cr=Cranial, Cd=Caudal.

Thoracic ultrasonography revealed increased pericardial sac thickness with an accumulation of exudates. The pericardial exudates varied from hyperechogenic contents representing fibrinous pericarditis (36.4%) (Figure-2a), hypoechogenic contents with hyperechogenic fibrin strands free inside the pericardium representing fibrino-suppurative pericarditis (30.9%) (Figure-2b), and anechogenic to hypoechogenic contents representing suppurative pericarditis (32.7%) (Figure-2c). The atria and ventricles appeared compressed. The pleural effusion, as one of the most noticed complications secondary to TP (58.2%), appeared as anechogenic exudates with distal enhancement (Figure-2d). In addition, lung consolidation was reported in 9.1% of the affected cows and represented by hyperechogenic foci with distal comet tail artifacts and loss of the characteristic reverberation artifacts of the normal lung.

**Figure-3:**
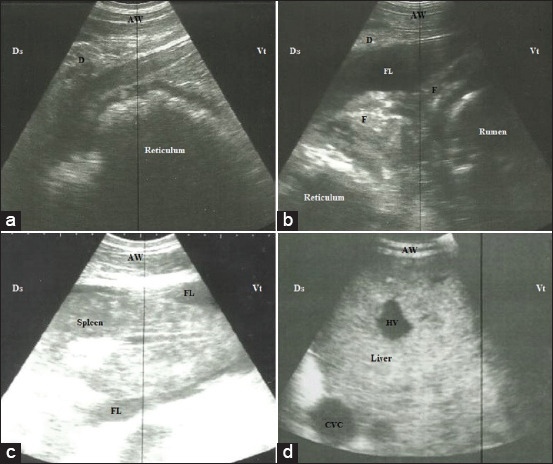
Ultrasonographic imaging of the abdomen of three cattle suffered from congestive heart failure due to traumatic pericarditis. (a) Corrugated reticular wall with separation of its layers in relation to the diaphragm when scanned at 6^th^ intercostal space (ICS). (b) Reticular adhesion with the rumen due to accumulation of fibrin threads between the reticulum and the diaphragm when scanned at 7^th^ ICS. (c) Splenomegaly with ascites, note the rounded ventral angle when scanned at the level of 7^th^ ICS. (d) Hepatomegaly with round-shaped CVC when scanned at 11^th^ ICS. AW=Abdominal wall, D=Diaphragm, F=Fibrin, FL=Ascetic fluid, HV=Hepatic vein, CVC=Caudal vena cava, Cr=Cranial, Cd=Caudal.

The abdominal ultrasonography revealed ascitic fluid inside the abdomen represented by anechogenic fluid in 52.7% of the affected cows. The reticulum was involved in all affected animals. The shape of the reticular wall varied from corrugation (49.1%) (Figure-3a), reticular adhesions with its neighboring structures (38.2%) (Figure-3b), and reticular abscess (12.7%). The reticular abscess appeared as a circumscribed hyperechogenic capsule with hypoechogenic to hyperechogenic content between the reticulum and the abdominal wall. The reticular contractions were reduced to be 2, 1, and 0/3 min that were found in 38.2%, 34.5%, and 27.3% of the animals, respectively. Splenomegaly was reported in 9.1% of the affected cows with slightly round ventral end (Figure-3c), while hepatomegaly with loss of acute angle was reported in 94.5% of the affected cows. The diameter of the PV and CVC was increased in comparison to the control group. The CVC lost its characteristic shape and became round or oval in cross-section (Figure-3d).

#### Pleurisy

**Figure-4:**
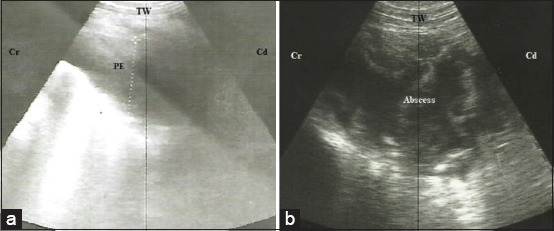
Ultrasonographic imaging of the thorax of two cattle with congestive heart failure. (a) Pleural exudate accumulation inside the pleural cavity. (b) Mediastinal abscess at the mediastinal region represented by heterogeneous echogenic contents with echogenic capsule. TW=Thoracic wall, PE=Pleural exudate, Cr=Cranial, Cd=Caudal.

Thoracic ultrasonography revealed pleural effusion represented by the presence of anechoic fluid within the pleural sac (Figure-4a). Suppurative pericarditis was reported in 28.6% of the affected cases. Interestingly, the heart and pericardium appeared normal in 71.4% of the affected cows. Pleural effusion and lung consolidation appeared in all the affected cows.

The ultrasonography of the abdomen revealed ascitic fluid in 14.3% of the affected cows. The reticular wall appeared normal, but its contractions were decreased to be 2 and 1/3 min and were reported in 71.4% and 28.6%, respectively. Hepatomegaly was reported in 57.2% of the affected cows. The diameter of the CVC and PV was increased than the control group. The spleen had a normal ultrasonographic appearance.

#### Mediastinal abscess

Thoracic ultrasonography revealed a large circumscribed hyperechogenic capsule with hypoechogenic to hyperechogenic contents in the mediastinal region (Figure-4b). Suppurative pericarditis was reported in only 40% of the affected cases. Interestingly, the heart and pericardium appeared normal in 60% of the affected cows. Pleural effusion and lung consolidation appeared in 40% of the affected cows.

Abdominal ultrasonography revealed ascitic fluid in 20% of the affected cows. The reticular wall appeared normal, but its contractions were decreased to be 2 and 1/3 min in 80% and 20%, respectively. Hepatomegaly was recorded in 60% of the affected cows. The diameter of the CVC and PV was increased than the control group. The spleen had a normal ultrasonographic appearance.

### Abdominocentesis and thoracocentesis

Ultrasonographic-guided abdominocentesis and thoracocentesis of 12 cows affected by TP revealed the presence of pleural and peritoneal transudate. However, thoracocentesis of two cows affected by pleurisy and three cows affected by mediastinal abscess revealed inflammatory exudate and purulent exudate, respectively.

## Discussion

CHF in cattle is one of the most important challenges in the veterinary field for the clinicians that occur when the heart fails to maintain the circulatory need of the body. The prognosis of such conditions is mostly poor and the animals are advised to be slaughtered or euthanized [22]. Pericardial, myocardial, and endocardial diseases were the most recorded causes of CHF in cattle as well as cardiac tumors and congenital defects [23]. In the present study, TP was the most recorded etiology of CHF, and for a lesser extent pleurisy as well as mediastinal abscess. TP as a causative disease of CHF agrees with that previously reported in cattle [24] and buffaloes [25]. Besides, there are several reports of pleural or lung-related pericarditis in cattle [9].

Regarding clinical findings, jugular engorgement, brisket and/or intermandibular edema, and abnormal heart sounds were of the most recorded clinical signs of CHF in cows. Similar findings have been previously reported in cattle [22] and buffaloes [25] affected by CHF. Cardiac compression with pericardial exudate and elevated hydrostatic pressure result in jugular distension and brisket edema [22].

In TP affected cows, most of the animals were reported with normal rectal body temperature (58.2%), while only 25.4% of the affected cases had a fever. Moreover, 16.4% of cases were reported with a subnormal temperature that might be attributed to the severity of the disease [22]. These results are in contrast with Radostits *et al*. [26] where they reported that fever was one of the main signs of TP in cattle and in agreement with Braun *et al*. [22] who reported that 16 of 28 cattle with fever and 3 of 28 with subnormal temperature. On the other hand, 71.4% and 60% of animals suffered from pleurisy and mediastinal abscess were reported with fever, respectively. Tympany was reported in 45.5% of the TP affected cows. This might be due to the penetration of the foreign body to the reticulum [16,24,27].

Muffled heart sounds were reported in all animals affected by pleurisy and mediastinal abscess as well as 70.9% of TP affected cows. These results are in parallel with that previously reported in cows affected by TP [22]. They reported that muffled sounds of the heart were a common symptom of pericarditis and in pleuropneumonia-affected cattle [21]. Tachycardia was reported in 35 cases out of 55 cows with TP and all cases affected by mediastinal abscess as well as five cases out of seven cows with pleurisy. During cardiac disease, multiple compensatory mechanisms are established, the first of which is tachycardia [28]. Tachycardia specificity for heart disease diagnosis is considered low because many non-cardiac diseases can induce tachycardia [29].

Abnormal lung sounds were reported in 58.2%, 100%, and 40% of the cows suffering from TP, pleurisy, and mediastinal abscess, respectively. This might be due to lung involvement secondary to penetrating foreign body in TP cases [16] and spreading infection in pleurisy and mediastinal abscess affected cases [21]. Dyspnea and cough were also common clinical signs in all cases with pleurisy. Furthermore, they were reported in 40% of the affected cases by mediastinal abscess as well as in 58.2% and 25.5% for dyspnea and cough, respectively, in affected cases by TP.

The penetration of foreign metallic bodies is the most common cause of TP in cattle [4,5]. The use of a metal detector for the diagnosis of TP is, therefore, of great importance which demonstrating positive results in 48 cases of 55 cows with TP. Similar results were reported by Braun *et al*. [22] where not all TP affected animals gave positive results to foreign body tests. Positive pain tests were reported in 63.6%, 100%, and 40% of affected cases by TP, pleurisy, and mediastinal abscess, respectively. These results might be due to abdominal and chest pain in the affected animals [22,24,29]. Arched back was reported in 63.6% of the affected cases by TP only, while recumbency was reported in 12.7% and 14.3% of cases affected by TP and pleurisy, respectively.

Many of the clinical findings reported in this study might be sensitive but not specific for diagnosis of TP, pleurisy, or mediastinal abscess. Therefore, the ultrasonographic examination of the cows enrolled in this study is a trusted non-invasive method [5,6,13,27,30]. They reported that ultrasonography is the most reliable, safe, and rapid aid for the diagnosis of thoracic and abdominal disorders in animals. Ultrasonographic examination revealed the involvement of the heart as the primary cause of CHF in all cases of TP. On the other hand, the heart was secondarily involved as a result of pleurisy or mediastinal abscess in a percentage of 28.6% and 40% of the affected cases, respectively.

Regarding the relationship between CHF and the other body organs, pleural effusion was reported in 58.2% as one of the most common complications secondarily to CHF in TP affected cows. These results were in agreement with the previous studies [31]. On the other hand, the pleural effusion and lung consolidation were reported in all cases affected by pleurisy. Moreover, the pleural effusion and lung consolidation were reported in 40% of the mediastinal abscess affected cows.

Reticular involvement was reported in all animals suffering from TP. The spleen was rarely involved in CHF. Only 7.5% of the affected cows reported with splenomegaly that was reported only in TP affected animals. This might be attributed to its position near to the reticulum [18,31].

Hepatomegaly secondary to CHF was reported in 88.1% of the affected animals. This might be due to cardiac insufficiency, and the blood returns to the CVC and the PV in the liver resulting in hepatic congestion and hepatomegaly [16,24,25]. Hepatomegaly is one of the common ultrasonographic findings in TP affected animals [13]. The liver angle became round or slightly acute in most animals with CHF. The diameter of the PV and CVC was increased than the normal control animals. The CVC lost its characteristic triangular-shaped and became oval to round. Ascites was reported in 52.7% of the TP affected cases, while for pleurisy and mediastinal abscess were reported in 14.3 and 20%, respectively.

## Conclusion

From the results of the present study, TP was the prevalent etiology of CHF in cattle. In addition, pleurisy and mediastinal abscess were recorded as extracardiac causes. The clinical findings of CHF are a good indicator of the diagnosis but are not confirmatory. Ultrasonography could confirm the diagnosis and recognize the associated etiology. Therefore, it is considered an appropriate diagnostic tool for thoracic and abdominal diseases when compared to the clinical findings.

## Authors’ Contributions

All authors designed, planned, drafted and revised the manuscript. MA, ME, and SAE contributed to clinical and ultrasonographic examinations. All authors read and approved the final manuscript.
